# Increased Adherence and Expression of Virulence Genes in a Lineage of *Escherichia coli* O157:H7 Commonly Associated with Human Infections

**DOI:** 10.1371/journal.pone.0010167

**Published:** 2010-04-21

**Authors:** Galeb S. Abu-Ali, Lindsey M. Ouellette, Scott T. Henderson, David W. Lacher, James T. Riordan, Thomas S. Whittam, Shannon D. Manning

**Affiliations:** 1 Microbial Evolution Laboratory, National Food Safety & Toxicology Center, Michigan State University, East Lansing, Michigan, United States of America; 2 Division of Molecular Biology, Center for Food Safety and Applied Nutrition, U.S. Food and Drug Administration, Laurel, Maryland, United States of America; 3 Department of Pediatrics and Human Development, Michigan State University, East Lansing, Michigan, United States of America; National Institutes of Health, United States of America

## Abstract

**Background:**

Enterohemorrhagic *Escherichia coli* (EHEC) O157:H7, a food and waterborne pathogen, can be classified into nine phylogenetically distinct lineages, as determined by single nucleotide polymorphism genotyping. One lineage (clade 8) was found to be associated with hemolytic uremic syndrome (HUS), which can lead to kidney failure and death in some cases, particularly young children. Another lineage (clade 2) differs considerably in gene content and is phylogenetically distinct from clade 8, but caused significantly fewer cases of HUS in a prior study. Little is known, however, about how these two lineages vary with regard to phenotypic traits important for disease pathogenesis and in the expression of shared virulence genes.

**Methodology/Principal Findings:**

Here, we quantified the level of adherence to and invasion of MAC-T bovine epithelial cells, and examined the transcriptomes of 24 EHEC O157:H7 strains with varying Shiga toxin profiles from two common lineages. Adherence to epithelial cells was >2-fold higher for EHEC O157:H7 strains belonging to clade 8 versus clade 2, while no difference in invasiveness was observed between the two lineages. Whole-genome 70-mer oligo microarrays, which probe for 6088 genes from O157:H7 Sakai, O157:H7 EDL 933, pO157, and K12 MG1655, detected significant differential expression between clades in 604 genes following co-incubation with epithelial cells for 30 min; 186 of the 604 genes had a >1.5 fold change difference. Relative to clade 2, clade 8 strains showed upregulation of major virulence genes, including 29 of the 41 locus of enterocyte effacement (LEE) pathogenicity island genes, which are critical for adherence, as well as Shiga toxin genes and pO157 plasmid-encoded virulence genes. Differences in expression of 16 genes that encode colonization factors, toxins, and regulators were confirmed by qRT-PCR, which revealed a greater magnitude of change than microarrays.

**Conclusions/Significance:**

These findings demonstrate that the EHEC O157:H7 lineage associated with HUS expresses higher levels of virulence genes and has an enhanced ability to attach to epithelial cells relative to another common lineage.

## Introduction

Enterohemorrhagic *Escherichia coli* (EHEC) O157:H7 contributes to many food and waterborne outbreaks as well as sporadic cases of enteric disease [1] that manifests as diarrhea, hemorrhagic colitis, or hemolytic uremic syndrome (HUS). Up to 60 deaths can result due to EHEC O157:H7 infection each year, which yielded a total cost of $405 million in 2003 in the U.S. alone [2]. The hallmarks of EHEC O157:H7 pathogenesis are attaching and effacing (A/E) lesions on the intestinal mucosa, and Shiga toxin (Stx)-mediated destruction of microcirculatory blood vessel endothelia. A/E lesions on epithelial cells present as actin pedestals that abut the intimately attached bacterium and as effacement of the absorptive microvillar brush border. This mode of colonization is mediated by the bacterial adhesin intimin, its translocated receptor (Tir), and several effectors [3], which are translocated into the host cell by a type three secretion system (TTSS) encoded by the locus of enterocyte effacement (LEE) pathogenicity island. The production of Stx (Stx1, 2 and variants) is coupled with phage lysis and, following receptor-mediated endocytosis, results in enzymatic inactivation of the eukaryotic 28S ribosomal subunit and host cell death [4].

While these virulence traits are shared between EHEC O157:H7 strains, the spectrum of clinical disease is broad, which may be due, in part, to genetic variation and differences in bacterial characteristics among strains. A prior single nucleotide polymorphism (SNP) genotyping study, for example, observed a high level of genetic diversity among >500 EHEC O157 strains and close relatives, which clustered into nine phylogenetically distinct clades comprising 39 SNP genotypes [5]. Remarkably, individuals with HUS were significantly more likely to be infected by an O157:H7 strain belonging to one lineage (clade 8), while a phylogenetically distinct lineage (clade 7) was associated with less severe disease [5]. These findings led to the hypothesis that EHEC O157 lineages vary in their ability to cause clinical illness. Although the biological basis behind this hypothesis is not known, it has been suggested that differences in gene content may be important as well as the presence of specific virulence characteristics, particularly the Shiga toxins [6], [7], [8]. Indeed, several studies have linked EHEC O157:H7 strains producing only Stx2 with severe post-infection sequelae [6], [9], [10], while other studies have identified associations with Stx2c alone [7] or in combination with Stx2 [8], [11].

Because of the conserved nature of most EHEC O157:H7 virulence genes, however, it is also possible that variable expression of genes that are shared between strains can cause differences in the ability to cause severe disease. A prior study of two divergent EHEC O157:H7 strains, which were implicated in the 2006 North American spinach outbreak (clade 8) [5] and the 1996 Sakai, Japan, outbreak (clade 1) [12], demonstrated that the spinach outbreak strain had an enhanced ability to colonize epithelial cells and express virulence genes following cell exposure [13]. These findings were corroborated by the observed differences in the ability of both outbreak strains to cause severe disease in germ-free mice, with the Sakai strain causing minimal histopathological damage [14]. Another study of bovine-derived EHEC O157:H7 strains found that the genotype (clade 8) associated with human infections had upregulation of key virulence genes compared to the genotype that predominates in bovines (clade 7), which had upregulation of stress fitness genes [15]. While these studies demonstrated clear differences in the gene expression profiles among a small sample of clade 8 strains (n = 4) relative to clades 1 (n = 1) and 7 strains (n = 4) from two sources, screening a larger sample of strains from divergent and clinically relevant lineages is necessary to determine why some lineages cause more severe disease than others.

In this study, genome-wide expression patterns were analyzed from 24 clinical strains of clade 8 (n = 12) and clade 2 (n = 12), the two predominant, but phylogenetically divergent, EHEC O157 lineages associated with clinical infections in Michigan between 2001 and 2006 [5]. Following exposure to epithelial cells, strains were examined for clade-specific differences in virulence gene expression and in the ability to colonize the epithelium. Within-clade differences among strains with varying Stx genotypes were also investigated. Overall, this study enhances our understanding of the functional differences between EHEC O157 lineages that are important for infection and highlights the importance of studying microbial pathogenesis in a phylogenetic context.

## Results

### O157:H7 interaction with epithelial cells

**Figure 1:**
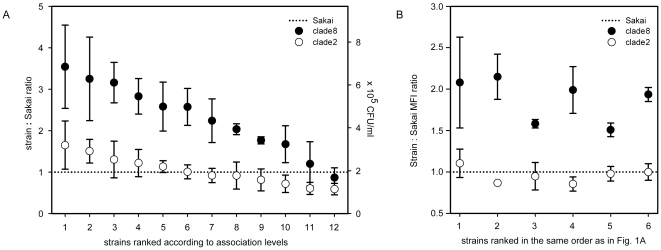
Association of 24 O157:H7 strains with MAC-T cells [Panel A]. Association levels of each strain were expressed relative to Sakai. Plotted on the ordinate are association ratios of test strain to Sakai (*y*-left), as well as CFU/ml plate counts (*y*-right). The symbols indicate the mean ± SD of three separate experiments. The dotted line represents the association level of Sakai. **Flow-cytometric quantification of association of 12 O157:H7 strains with MAC-T cells [Panel B].** Clade 8 (n = 6) and 2 (n = 6) strains with highest association levels, determined by plate counting, were ranked in the same order as in panel A, and differences in association were expressed as the ratio of MFI of strain-infected MAC-T cells and the MFI of Sakai-infected MAC-T cells. Bars represent the mean ± SD of two separate experiments. The dotted line represents the association level of Sakai.

Association assays were performed to quantify the ability of clade 8 (n = 12) and clade 2 (n = 12) strains to associate with MAC-T cells. Following 1 h of incubation, all strains were associated with MAC-T monolayers; however, clade 8 strains associated with greater numbers than clade 2 strains (**Figure 1** **, Panel A**). Specifically, clade 8 strains demonstrated a 2.3±0.2 fold higher association with MAC-T cells than clade 2 strains (*P* = 0.0001). This finding was confirmed by flow cytometric analyses of the six strains of both clades with the highest association levels (*P* = 0.00001) (**Figure 1** **, Panel B**). Invasion assays demonstrated no difference (*P* = 0.22) in the ability of all 24 strains to invade MAC-T cells regardless of clade, and furthermore, levels of invasion were an order of magnitude lower than association levels (**Supplemental Figure S1**). Together, these data indicate that adherence of EHEC O157:H7 clade 8 strains to MAC-T epithelial cells is increased compared to clade 2 (**Supplemental Figure S2**).

### Gene expression analyses

**Figure 2:**
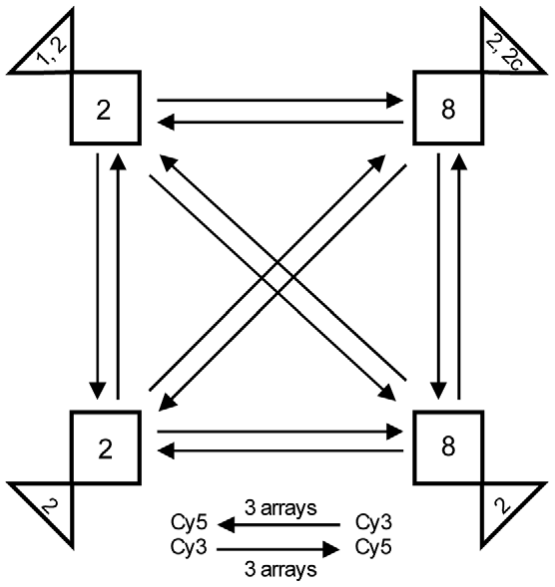
Microarray hybridization scheme. 24 O157:H7 strains were divided into 4 groups based on clade (squares) and Stx genes (triangles). The six strains of each group were considered as individual biological replicates of the particular group (n = 6). Randomized hybridizations were performed between groups so that six strains from one group were randomly matched to six strains from another group, with dye-swaps. Six hybridizations were performed between any two groups; for each of the six hybridizations, cDNAs belonging to a different pair of strains were compared. In total, 36 hybridizations were performed.

**Figure 3:**
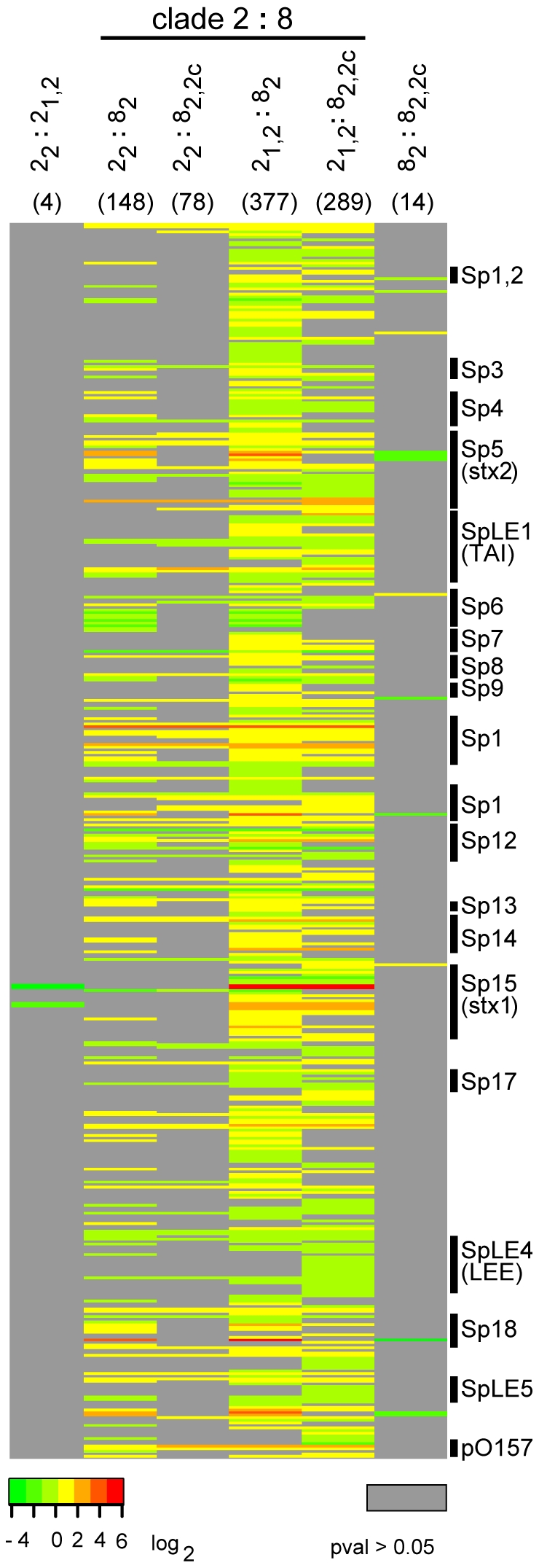
Pairwise contrast analysis of differentially expressed genes between four groups of O157:H7 strains. Each column represents log_2_ differences in expression between 2 groups (clade*_stx_*). In parenthesis atop each column is the number of genes indicated to be significantly differentially expressed between the 2 groups compared. Color green indicates increased expression in the denominator group. Genes were sorted by chromosomal positions and the heat map was generated in R (‘gplots’ package version 2.3.2). Sp – Sakai prophage, SpLE – Sakai prophage-like element, pO157 – EHEC plasmid, TAI – Tellurite resistance and adherence conferring island.

To determine whether EHEC O157:H7 gene expression profiles are similar among strains of the same clade or strains with the same Stx profile, regardless of clade, we analyzed transcriptomes of 24 strains classified into four groups based on clade and Stx profile (**Figure 2**). In all, 363 genes were differentially expressed (*P*<0.05) among the four groups. A dendrogram based on group column means clustered the groups according to clade (**Supplemental Figure S3**), which suggested that strains from the same clade had similar transcriptional profiles. Pairwise contrast analysis of expression values between each pair of strain groups indicated that the number of differentially expressed genes among inter-clade groups was between 20 and 94 times higher than among intra-clade 2 groups and between 6 and 27 times higher than among intra-clade 8 groups (**Figure 3**). Four genes, including *stx1A, stx1B*, a putative prophage repressor, and an unknown gene present on the Stx1-prophage (Sakai prophage (Sp)-15) [12], were differentially expressed within clade 2. Similarly, only 14 genes were differentially expressed within clade 8. Most of these genes are phage borne and of unknown function; however, the microarrays lack the 75 ORFs specific for the 2851 Stx2c-harboring phage [16], a component present in 6 of the clade 8 strains, but in none of the clade 2 strains. Analysis of the ‘*Clade:Stx*’ interaction effect on gene expression differences among O157:H7 strains did not indicate that the expression of any gene in either clade was influenced by the *stx* profile.

The comparison of transcription profiles between clades 8 and 2, irrespective of Stx profiles, indicated a significant difference in the expression of 604 genes; 186 had a ≥1.5 fold change (**Supplemental Table S1**), above which the difference in expression is considered meaningful [17]. A total of 265 of the 604 genes encode hypothetical proteins of unknown function, and 316 of the 604 genes are phage borne. A comparison to the published EHEC O157:H7 genome sequences identified 53 Sakai ORFs in clade 8 and four in clade 2, out of the 316, with little or no homology to the respective genome sequences and were therefore not evaluated further. Relative differences in gene expression of 16 O157:H7 virulence determinants (**Table 1**) were examined more thoroughly by qRT-PCR. Although none of the genes tested by qRT-PCR had a >2-fold change via microarrays, these microarray data are comparable to those generated in a prior study among single O157:H7 strains grown in the presence and absence of epithelial cells [18]. It is possible, however, that the degree of intra- and inter-clade variability resulting from our analysis of 24 different strains may have dampened the effect.

**Table 1:** Relative differences in gene expression between clades 8 and 2, as detected by microarrays and qRT-PCR.

*gene*	ECs^a^	protein	Microarray		qRT-PCR	
			fold change^b^	*P*	fold change^c^	E^d^
*espA*	4556	translocator, LEE4	2.0	0.022	5.90±0.65	2.00
*espB*	4554	translocator, LEE4	2.0	0.005	3.55±1.00	2.02
*Tir*	4561	receptor, LEE5	1.8	0.008	2.43±0.39	1.99
*eae*	4559	intimin, LEE5	1.6	0.009	2.07±0.52	1.98
*escN*	4568	structural, LEE3	1.5	0.008	2.06±0.12	1.96
*sepZ*	4571	effector, LEE2	1.4	0.075	1.72±0.08	2.12
*ler*	4588	regulator, LEE1	1.3	0.091	1.09±0.16	2.07
*stx2A*	1205	Stx 2 subunit A	1.8	0.001	5.19±0.55	2.06
*stx2B*	1206	Stx 2 subunit B	2.0	0.000	5.39±0.16	2.01
*q^e^*	1203	Q antiterminator	1.8	0.000	2.90±0.13	1.99
*hlyA*	pO157	EHEC hemolysin A	1.5	0.006	5.04±0.49	2.04
*toxB*	pO157	Toxin B	1.3	0.001	2.64±0.27	1.96
*stcE/tagA*	pO157	StcE metalloprotease	1.5	0.060	2.34±0.06	2.01
*grlA*	4577	GrlA regulator, LEE	1.5	0.025	3.45±0.11	2.02
*rpoS*	3595	sigma factor 38	−1.7	0.009	−2.99±0.60	2.21
*gadX*	4396	GadX regulator	−1.1	0.042	−4.70±1.29	2.08

a. *E. coli* O157:H7 Sakai chromosomal gene numbers [12].

b. Fold change differences between clade 8 (n = 12) and clade 2 (n = 12) strains; positive values were upregulated in clade 8.

c. mean ± standard deviation of relative fold change differences in gene expression between clade 8 (n = 6) and clade 2 (n = 6) strains; positive values were upregulated in clade 8.

d. mean reaction efficiencies of 12 O157:H7 strains.

e. qRT-PCR fold change in expression of the *q* antiterminator is based on clade 8 (n = 3) and 2 (n = 3) strains that carry only *stx2* genes.

### Locus of enterocyte effacement (LEE) island genes

**Figure 4:**
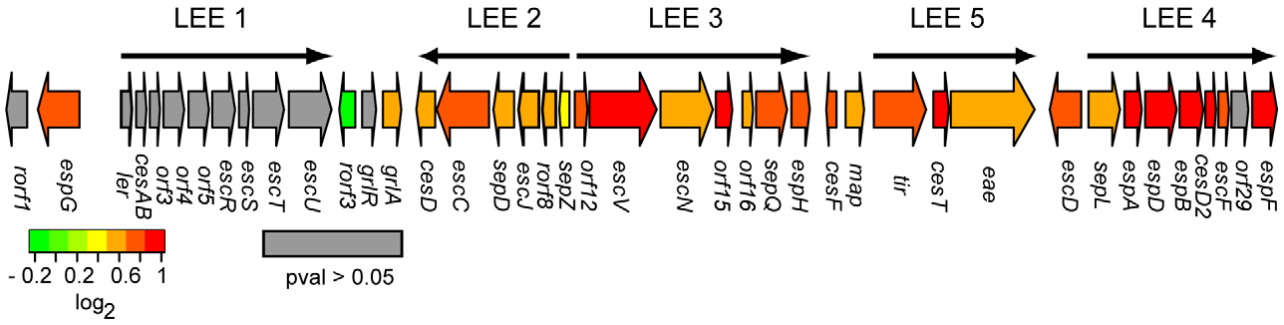
LEE expression differences between clades 8 and 2. For clade 8 to 2 ratios, expression data from the ‘Clade effect’ analysis was used to generate the heatmap in R (‘gplots’ package version 2.3.2) that was then fitted to a graphic representation of the genetic organization of the LEE island, adopted from [62]. Color red indicates increased expression in clade 8.

Twenty-nine LEE genes were upregulated in clade 8 relative to clade 2 (**Supplemental Table S1 and** **Figure 4**), with a subtle decrease in fold change from the LEE4 to the LEE1 operon. Apart from *sepL*, transcription was highest in LEE4 genes (1.92±0.13-fold), especially for the *espADB* polycistron that encodes the molecular syringe of the TTSS. Expression of LEE5 was slightly lower (1.72±0.13-fold) followed by LEE3 (1.66±0.14-fold) and LEE2 (1.50±0.09-fold), which encode the membrane-bound TTSS components. LEE1 genes, as well as *rorf1*, *grlR* and *orf29* lacked significant differential expression (*P*>0.05). To validate the LEE microarray data, mRNA levels of *ler*, *sepZ*, *escN*, *espA*, *espB*, *tir*, and *eae*, which were selected as representatives of each of the LEE operons, were measured by qRT-PCR (**Table 1**). Microarrays also detected significant expression of 13 non-LEE effector genes, 11 of which were upregulated in clade 8 (Table S2); these genes are located on various prophages that are dispersed throughout the O157:H7 chromosome and code for proteins exported by the TTSS [3], [19]. Collectively, these data demonstrated upregulation of the LEE island in clade 8 versus clade 2 strains preceding attachment to epithelial cells.

### Shiga toxin genes

**Figure 5:**
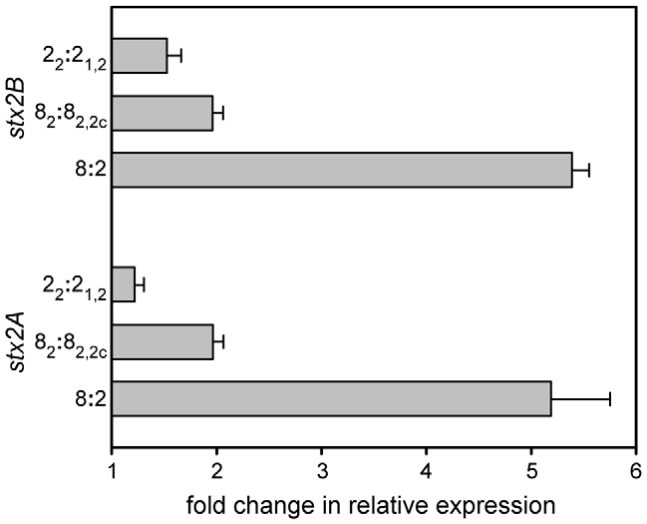
Differences in expression of Stx2 genes within and between clades, as determined by qRT-PCR. Labels on the ordinate represent clade*_stx_* profiles of examined strains. Differences in expression are plotted as average fold change ratios, with standard deviations, of clade 8 (n = 6) to clade 2 (n = 6) strains; for within clade comparison, averages of 3 strains per group are compared.

Stx1 genes, *stx1A* and *stx1B*, were upregulated in the clade 2 strains that harbor the Stx1-converting phage (**Supplemental Table S1**), a result that was expected given that virtually all clade 8 strains examined in a prior study lacked *stx1* [5]. By contrast, clade 8 strains demonstrated increased expression of *stx2A* and *stx2B* compared to clade 2; this result was also confirmed by qRT-PCR (**Table 1**). Furthermore, transcription of *stx2A* and *stx2B* was higher in both clades for strains lysogenized exclusively with the Stx2-converting phage compared to strains with either Stx2 and Stx2c, or Stx2 and Stx1 phages (**Figure 5**). The phage borne antitermination gene *q* (ECs1203), located upstream of *stx2,* was also upregulated in clade 8. While qRT-PCR confirmed the increased expression of *q* in clade 8 (**Table 1**), it also detected 2-fold higher *q* transcript levels in strains possessing two Stx phages relative to strains with only the Stx2-phage. This result likely reflects sequence similarities among *q* antiterminators of different Stx-phages, which could not be resolved by sequence-specific primers. qRT-PCR results from strains of both clades with only *stx2* confirmed the upregulation of *q* in clade 8 (**Table 1**).

Because sequence divergence of *q* has been associated with variation in transcription of *stx2* [20], a sequence alignment of a 2.5 Kb fragment from the *q* gene through *stx2B* was conducted among the four O157:H7 genome strains. Through this analysis, four SNPs were identified in the clade 8 genome, while none were detected in the remaining strains. These four SNPs were found in the: i) *pS* tRNA promoter, ii) 2 bp deletion between *tRNA-Ile* and the first *tRNA-Arg*; iii) first *tRNA-Arg*; and iv) *stx2A* coding region. DNA sequences important for *stx2* transcription efficiency [21], including the *q* antiterminator, *pR*' *stx2* promoter, *qut* site, *tR*'*1* and *tR*'*2* terminators, and of the *stx2B* subunit, were identical in all four genome strains.

### Regulators

The expression of *rpoS*, *grlA*, and *gadX*, which have been shown to regulate the LEE island [22], were significantly different between clades 8 and 2 (**Table 1**). Upregulation of the sigma factor 38 (*rpoS*) in clade 2, with the concurrent down-regulation of the LEE in clade 2, supports the prior observation that RpoS negatively influences LEE gene expression [23], [24]. Microarrays demonstrated a statistically significant increase in *gadX* transcription in clade 2, though a much greater increase with a higher standard deviation was observed using qRT-PCR, suggesting variation among clade 2 strains (**Table 1**). The LEE-encoded positive regulator, *grlA*, was also upregulated in clade 8 strains. Apart from its stimulatory effect on LEE, *grlA* also mediates expression of non-LEE encoded effectors [25].

### Plasmid borne virulence genes

Two adhesion-associated pO157-borne genes, *toxB* and *tagA/stcE*, were upregulated in clade 8 (**Table 1**). The ToxB protease was demonstrated to post-transcriptionally stimulate expression of LEE4 proteins and facilitate adhesion to epithelial cells [26], [27]. Although the increase in transcription of *tagA/stcE* in clade 8 was only marginally significant (*P* = 0.06) via microarrays, qRT- PCR detected more than 2-fold upregulation in clade 8 strains (**Table 1**). Differential expression of the *etp* operon, the plasmid-encoded type II secretion system that directs the export of StcE, was not detected. Finally, significant upregulation of *hlyA* (**Table 1**), which encodes the pore-forming RTX (repeats in toxin) EHEC hemolysin A (EHEC-HlyA), was observed.

## Discussion

Prior studies involving transcriptional profiling of *E. coli* O157:H7 strains before and after exposure to eukaryotic cells have increased our knowledge of the molecular events underpinning O157:H7 infection of the host cell [18], [28], [29]. However, because of the significant genetic diversity in the extant O157:H7 population [5], [30], [31], we sought to determine whether strains of diverse lineages have similar adherence capabilities and responses to host cell exposure. Indeed, our prior study using a tissue culture model for O157:H7-epithelial cell challenge under conditions that mimic the pathogen-host interaction, has demonstrated phenotypic and transcriptional differences among the divergent Sakai (clade 1) and spinach (TW14359; clade 8) outbreak genome strains [13]. These findings were corroborated by the variation in histopatholology observed following infection with both strains in germ-free mice [14]. Here, we applied the same methodology to a set of 24 clinically relevant O157:H7 strains from two divergent lineages, and have observed variation in adherence levels to epithelial cells as well as global gene expression profiles. By sampling strains that differ in Stx profile, we were also able to assess the variation associated with different Stx genotypes within and between O157:H7 clades.

Consistent with previous findings [13], there was no variation in the degree of epithelial cell invasion between strains of the two different lineages. Strains belonging to clade 8, however, demonstrated an increased ability to adhere to epithelial cells, suggesting that clade 8 strains are better than clade 2 strains at initiating colonization, the first step in disease pathogenesis. Alternatively, it is also possible that clade 8 strains begin expressing genes important for adherence earlier than clade 2 strains. Conducting a similar study at multiple time points, however, is required to address the latter possibility. The adherence capacity of each strain was also determined by Stx profile, as expression of Stx2 was previously suggested to contribute to adhesion of O157:H7 to HEp-2 cells [32]. In this study, adherence differences between strains were not influenced by any particular *stx* profile, but rather, were clade-specific, as increased adherence was observed in clade 8 relative to clade 2 even among strains with only *stx2*.

Based on the observed differences in global gene expression between clades, there are several possible explanations for the enhanced ability of clade 8 versus clade 2 strains to adhere to epithelial cells. In fact, a total of 29 LEE genes were differentially expressed by microarrays as were several pO157 and global regulator genes involved in attachment to host cells; 16 of these genes were confirmed by qRT-PCR.

First, overall expression of LEE island genes, which are critical for the development of A/E lesions and disease pathogenesis [19], was higher in clade 8. Specifically, relative expression was highest in genes encoding the needle complex of the TTSS (EspADB) and in LEE-encoded effector genes. Transcription of LEE1, which encodes the basal membrane-bound secretion machinery and the Ler regulator, however, was not significantly upregulated in clade 8. This is an unexpected finding because Ler can directly activate transcription of *grlRA*, LEE2, LEE3, LEE5, and LEE4, as demonstrated with electrophoretic mobility shift assays [33], [34], [35] and reporter gene transcriptional fusions [36]. Because assembly of the TTSS is sequential starting with membrane-bound components [19], and that O157:H7 polycistronic TTSS mRNAs are not degraded at the same rate [28], it is possible that transcription levels were determined at a moment of temporal overlap between the two clades. In other words, if LEE1 transcription in clade 8 strains was initiated before clade 2 strains, then LEE1 mRNAs may have degraded earlier, thereby concealing higher expression levels in clade 8. Alternatively, it is possible that LEE1 expression levels are similar between the two clades, but that other LEE operons are differentially expressed due to activation via alternative mechanisms or pathways. For example, mutation of *grlA* in *Citrobacter rodentium* has been shown to reduce expression of both LEE2 and LEE5 genes [25], while, in O157:H7, the GrlA regulator can be activated via the QseA transcriptional factor acting through an unknown intermediate [35]. In the latter pathway, it was also demonstrated that GrlA can then directly activate LEE2 and LEE4 genes [25], [35].

Another explanation for the differential expression of LEE island genes may involve the RpoS transcription factor, as *rpoS* was upregulated in clade 2. The contribution of RpoS to LEE expression, however, is conflicting, as both the activation [37] and repression of LEE [23] by RpoS have been reported. Our results support the finding that RpoS negatively influences LEE expression, which was demonstrated to occur via a regulatory cascade involving the repression of *ler* activators *pchABC*, by an unidentified factor [23]. Additionally, upregulation of *gadX* in clade 2, which was shown to inhibit LEE expression through downregulation of the plasmid-encoded Per regulator in enteropathogenic *E. coli* [38], may also be important. Interestingly, none of the other factors previously implicated in the transcriptional control of LEE [22], including H-NS, Pch, SdiA, QseA, ClpXP, IHF, and BipA, had significant differential expression between clades. Although this may be ascribed to the transient stability of regulator mRNA or inter-clade variation, additional studies are required to fully understand the complex regulatory circuits that govern LEE transcription among distinct clades. Whether differences between clades are attributable to SNPs, for example, which increase promoter efficiency or involve unidentified factors, is not known.

Upregulation of the pO157 plasmid genes, *toxB* and *tagA*/*stcE*, which were previously shown to be important for bacterial attachment, was also observed. ToxB is a partial homologue of lymphostatin (LifA), which is common among A/E *Enterobacteriaciae* and is associated with the inhibition of lymphocyte activation through inhibition of IL-2 interleukin activation [39]. Mutation of *lifA* in *C. rodentium* abolishes colonic inflammation in mice and leads to a significant reduction of colonization [40]. In O157:H7, ToxB is required for complete adherence, as a *toxB* mutation results in decreased adhesion to Caco-2 cells and reduced secretion of LEE-encoded factors, including EspA, EspB, EspD and Tir [26], [27]. Similarly, deletion of *tagA/stcE* results in decreased adhesion of O157:H7 to HEp-2 cells [41]. The TagA/StcE zinc metallo-protease is hypothesized to promote adherence by cleaving proteins in the glycocalyx and mucin layers atop the intestinal epithelium, thereby allowing O157:H7 to come into close contact with the intestinal mucosa [41]. While differential expression of *stcE* was insignificant in the microarray analysis, qRT-PCR detected a significant difference in *stcE* transcript levels between clades. Also, the qRT-PCR efficiencies indicate that sequence divergence among O157:H7 strains did not bias qRT-PCR results for the genes examined.

Perhaps the most important finding is the >5-fold increase in clade 8 transcription of Stx2, the key virulence determinant in the development of HUS [4], [42]. While it is unclear how a >5-fold increase in transcription specifically relates to disease, our prior study [13] comparing Stx2 transcription within TW14359 and Sakai before and after MAC-T exposure, demonstrated no difference in the levels of *stx2A* and *stx2B* expression by qRT-PCR. Therefore, we hypothesize that a >5-fold increase in transcription following MAC-T exposure among one group of phylogenetically similar strains relative to another group is biologically significant. This hypothesis does not, however, confirm that the prior epidemiological association identified between clade 8 strains and HUS [5] was due to variable Stx2 production. Consequently, comparing basal expression levels of *stx2A* and *stx2B* to levels expressed following exposure to other epithelial cell lines warrants further study.

Variable expression and production of Stx2 between divergent O157:H7 strains has been previously reported [13], [43], [44], although those bacterial components that contribute to increased expression are not fully understood. Stx2 transcription is dependent on prophage induction, which is principally initiated through the bacterial DNA damage response (SOS) pathway [45]. In this study, we did not observe differential expression of any SOS regulon genes. Therefore, it is unlikely that the activation of the *q* antiterminator and the subsequent upregulation of *stx2* expression in clade 8 is solely attributed to SOS-mediated amplification of lysogenic induction in clade 8 strains. Analysis of phage DNA sequences upstream of *stx2* in four O157:H7 genome strains of clades 1, 2, 3, and 8, demonstrated that sequence variation is not likely to play a role in the differential transcription of Stx2; no SNPs were identified in phage DNA regions known to influence RNA polymerase efficiency and *stx2* transcription [21]. This lack of variation was also noted in a previous study [46], as sequences were identical among several Stx-phages in the λ phage operator genes (O*_L_* and O*_R_*) that flank the *cI* repressor and are directly involved in the regulation of prophage induction. Other possibilities for the SOS-independent activation of Stx2 transcription include a high level of spontaneous phage induction in certain lineages or induction by unknown bacterial factors [43], a possibility that could not be addressed in the current study. Future work should therefore focus on comparing Stx2 expression levels, at multiple time points between isogenic hosts transduced with Stx2 phages from different O157:H7 clades. It is also important to note that *stx2* transcription was upregulated in strains of both clades 8 and 2 containing only the Stx2-phage, compared to strains that contain two Stx phages. This result is consistent with findings from a prior study [47], as the incorporation of two Stx2-phages into the K12 chromosome demonstrated a decrease in Stx2 production compared to K12 strains transduced with only one Stx2 prophage. This reduction was hypothesized to be mediated by the CI repressor of Stx-phages acting in trans, which can ultimately lead to reduced pathogenicity of the host strain [47]. Nevertheless, *stx2* expression was still significantly higher in clade 8 when strains with only *stx2* were compared among both clades.

This study also identified genes with no hypothesized role in bacterial attachment to be upregulated in clade 8 strains. One noteworthy example is the plasmid-borne *hlyA*. Antibodies to EHEC-HlyA have been detected in sera from convalescent HUS patients [48], and a cytotoxic effect of this pore-forming toxin on human endothelial cells has been demonstrated [49]. These observations suggest that EHEC-HlyA contributes to severe disease by destruction of the microcirculatory endothelium [49], the primary tissue affected in HUS [42]. Increased expression of *hlyA* could be attributed to GrlA, which was shown to stimulate transcription of *hlyA* [50] and was also upregulated in clade 8. Together with increased expression of Stx2, the key agent of endothelial cell damage, upregulation of EHEC-HlyA may partly explain the epidemiological association between clade 8 and HUS identified in a prior study [5].

In short, the data presented here confirm that subpopulations of O157:H7 are not only genetically diverse, but also have distinct phenotypic traits and transcriptional profiles following exposure to host cells. The increased expression of genes important for attachment among clade 8 versus clade 2 strains explains, in part, the enhanced ability of clade 8 strains to adhere to epithelial cells at a specific time point. One explanation for these observations is the presence of an unknown master “switch” that is responsible for activating the expression of virulence determinants. This possibility invites speculation that there may be distinct regulators, or regulatory cascades, of shared virulence genes among O157:H7 lineages. Future investigation, however, is warranted to determine this as well as assess how time since epithelial cell exposure affects virulence gene transcription and protein production between clades.

## Materials and Methods

### Bacterial strains and culture conditions

Twenty-four O157:H7 strains representing clades 8 and 2, as determined by SNP genotyping in a prior study [5], were evaluated. Clades 8 and 2 were chosen because they 1) represented the most prevalent clades; 2) are phylogenetically distinct; and 3) were implicated in HUS cases, although HUS was significantly more likely to be caused by clade 8 than clade 2 strains. Strains were also selected to represent individual SNP genotypes that constitute each clade as well as the *stx* combination (**Table 2**). The O157:H7 RIMD 0509952 (Sakai) strain [12] was used as a reference. Cultures were successively grown in LB broth, morpholino-propanesulfonic acid (MOPS) buffered minimal media (0.1% glucose, pH 7.4), and Dulbecco's Modified Eagle's Medium (DMEM) (pH 7.4, without phenol-red, 0.45% glucose, 0.37% NaHCO_3_), as described previously [13]. Log-phase O157:H7 cultures in DMEM, at cell concentrations of (5±1)×10^8^ CFU/ml and with a pH range of 7.09 to 7.20, were used in all experiments.

**Table 2:** Clade, Shiga toxin gene (stx) profile and source of O157 strains.

Strain Number	Strain Name	Clade	*stx*	Location of isolation	Date^a^	Clinical presentation^b^
TW08623	EK15	2	*1,2*	USA (WA)	2002	Diarrhea
TW10012	F6854	2	*1,2*	USA (PA)	1998	Unknown
TW04863	93-111	2	*1,2*	USA (WA)	1993	Diarrhea
TW10045	H2498	2	*1,2*	USA (CT)	1996	Unknown
TW11308	96M1006	2	*1,2*	Australia	1996	Bloody diarrhea
TW07961	DA-35	2	*1,2*	USA (OH)	1998	Unknown
TW11028	MI02-57	2	*2*	USA (MI)	2002	Bloody diarrhea
TW11029	MI02-1	2	*2*	USA (MI)	2002	Bloody diarrhea
TW14279	MI05-10	2	*2*	USA (MI)	2005	Bloody diarrhea
TW11037	MI02-68	2	*2*	USA (MI)	2002	Bloody diarrhea
TW11110	MI04-43	2	*2*	USA (MI)	2004	HUS
TW11185	MI01-29	2	*2*	USA (MI)	2001	Bloody diarrhea
TW14359	MI06-63	8	*2,2c*	USA (MI)	2006	Bloody diarrhea
TW02883	E32511	8	*2,2c*	Unknown	1991	HUS
TW08635	EK27	8	*2,2c*	USA (WA)	2002	HUS
TW07591	1∶361	8	*2,2c*	USA (MI)	1997	Diarrhea
TW08030	MT#9	8	*2,2c*	USA(MT)	2000	Bloody diarrhea
TW09189	MI03-72	8	*2,2c*	USA (MI)	2003	Bloody diarrhea
TW11032	MI02-55	8	*2*	USA (MI)	2002	Diarrhea
TW08609	EK1	8	*2*	USA (WA)	1999	Diarrhea
TW08610	EK2	8	*2*	USA (WA)	2001	Diarrhea
TW07937	DA-11	8	*2*	USA (MA)	1998	Bloody diarrhea
TW09098	MI03-35	8	*2*	USA (MI)	2003	Bloody diarrhea
TW14313	MI06-31	8	*2*	USA (MI)	2006	HUS
TW08264	Sakai	1	*1,2*	Japan	1996	Unknown

a. Year of strain isolation or deposition into the STEC Reference Center at Michigan State University.

b. Unknown refers to missing data; HUS, hemolytic uremic syndrome.

### Association and invasion assays

Bovine mammary epithelial cells (MAC-T) were cultivated as previously described [13]. MAC-T cells, which are commonly used in studies of adherent and invasive *E. coli*, were selected primarily because a prior study demonstrated no difference in the ability of EHEC O157:H7 strains to adhere to human epithelial cells (HEp-2) when compared to several primary intestinal epithelial cell lines of bovine origin [51]. The fluorescent actin staining (FAS) assay was used as a qualitative test [52] to ensure that a representative clade 2 strain had the ability to form A/E lesions on MAC-T cells and colonize in a ‘localized adherence’ pattern (**Supplemental Figure S4**). A representative clade 8 strain, TW14359, was tested previously [13].

Assays to quantify cell association, which is defined as the combination of adherence and invasion, were performed in 24-well plates as described [13]. Briefly, MAC-T cell monolayers were infected with bacterial cultures at a multiplicity of infection (MOI) of 500∶1. After 1 h of incubation (37 °C, 5% CO_2_), cells were washed with PBS, disrupted with 0.1% Triton X-100, plated on LB agar, incubated overnight (37 °C), and enumerated. For invasion assays, cells were washed with PBS 1 h post-incubation, incubated for 2 h with DMEM containing 200 µg/ml of gentamicin to kill extracellular bacteria, plated, incubated overnight, and enumerated [13]. The number of bacterial cells that had adhered was determined by taking the difference between the association and invasion numbers as described previously [51]. O157:H7 strains were assayed in triplicate and the experiment was repeated three times. The mean CFU/ml of each strain from three wells (biological replicate) was normalized against the mean CFU/ml of Sakai (clade 1), which was used as a reference. Normalized data were analyzed with a mixed ANOVA model where relative association  =  strain + replicate (strain) + error. The biological replicate was nested within the strain effect. Analysis was conducted using PROC MIXED (SAS version 9.1), and differences in association, adherence and invasion levels between clades were expressed as the mean ± SD of clade 8 relative to clade 2.

### Flow cytometry

Association assays were repeated and quantified using flow cytometry for the subset of clade 8 (n = 6) and clade 2 (n = 6) strains that demonstrated the highest association levels based on standard plate counts. Bacteria were labeled using the Vybrant® CFDA SE (carboxyfluorescein diacetate, succinimidyl ester) Cell Tracer Kit (Molecular Probes, Eugene, OR) following the manufacturer's recommendations. Briefly, 4 ml aliquots of O157:H7 cultures were centrifuged at 3200×*g*, 4 °C, for 10 min to pellet cells. Pellets were suspended in 2 ml of fresh DMEM, cultures were adjusted to a final CFDA SE concentration of 30 µM, and incubated for 20 min in the dark at 37 °C with gentle shaking. O157:H7 cultures were then centrifuged to remove excess dye, suspended in 4 ml of fresh DMEM, and incubated for 30 min to stabilize dye incorporation. After another centrifugation, pellets were suspended in 4 ml of fresh DMEM and used for association assays. After a 1 h incubation, wells were washed with PBS and MAC-T cells were detached by incubating with 0.5 ml of a trypsin solution (50 mg trypsin and 1 ml of 62 mM EDTA in 99 ml of PBS) for 10 min. Following addition of 0.5 ml of DMEM to each well, cell suspensions were centrifuged, and the pellets were suspended in 300 µl of buffer (0.1% sodium-azide and 1% fetal bovine serum in PBS) for FACS analysis using the Vantage flow cytometer (BD Biosciences, San Jose, CA). From each sample, 15,000 viable MAC-T cells were analyzed after setting a live cell gate based on the forward and side scatter profiles of uninfected MAC-T cells. Each strain was assayed in duplicate and the experiment was repeated twice. The mean fluorescence intensity (MFI) of test strain-infected MAC-T cells was normalized against the MFI of Sakai-infected MAC-T cells. The normalized MFI data were analyzed by the Student's *t*-test to determine statistically significant differences in association between clades.

### MAC-T challenge experiments, microarray hybridization and analysis

RNA was harvested from the 24 O157:H7 strains exposed to, but not attached to, MAC-T monolayers for 30 min, using a previously described *in vitro* model of O157:H7 exposure to MAC-T cells [13]. O157:H7 RNA extraction, cDNA synthesis, and dye-swap hybridization conditions were described elsewhere [13]; the microarray platform (Operon *E. coli* oligo set version 1.0.2) probes for 5978 ORFs from *E. coli* O157:H7 Sakai, O157:H7 EDL 933, and K12 MG1655, and for 110 ORFs from the pO157 plasmid [13]. Thirty-six dye-swap hybridizations were performed between four groups with six strains per group, according to a balanced double loop design [53] depicted in **Figure 2**. Strains were grouped based on clade*_stx_* profiles and each strain was considered an independent biological replicate of its group (n = 6). The six strains from each group were randomly hybridized with six strains from every other group; each hybridization compared a pair of strains that differed in either clade or *stx* profile, or both. The microarray data have been deposited in NCBI's Gene Expression Omnibus (http://www.ncbi.nlm.nih.gov/geo) and are accessible through accession number GSE20397.

Subsequent to local Lowess normalization [54], averaging of replicate probes and log_2_ transformation [55], the microarray data were fitted to a 2-factor mixed ANOVA model (*intensity*  =  *Array + Dye + Clade + Stx + Clade:Stx + Sample;* where the biological replicate (*Sample*) and array effects were considered random effects, while all other effects were considered fixed effects), using the MAANOVA package (version 0.98–8) [56] in R software (version 2.2.1). This model allows independent consideration of the effect of ‘*Clade*’ (clade divergence) and ‘*Stx*’ (*stx* type variation) parameters on differences in gene expression among O157:H7 strains, as well as their interaction (combined) effect (*Clade:Stx*). Overall differences in gene expression between groups were determined using the Fs test [57], followed by pair-wise contrasts to determine significant differential expression between each pair of groups [55]. Subsequently, the Fs statistic was estimated for the ‘*Clade*’ effect to determine significant differences in gene expression between clades 8 (n = 12) and 2 (n = 12), regardless of *stx* profile. The Fs statistic was also used to estimate the *‘Stx’* effect and the ‘*Clade:Stx*’ interaction effect to examine the combined effect of clade and *stx* type on differences in gene expression among O157:H7 strains. In other words, this analysis will determine whether the expression of any given gene among groups with different *stx* types is also dependent on clade. An interaction effect would be observed if expression estimates between strain groups clade8*_stx2_* and clade2*_stx2_* are different from expression estimates between strain groups clade8*_stx2,2c_* and clade2*_stx1,2_*. For every analysis, 1000 permutations of the data were performed to generate *P* values; estimates were considered significant if the *P* value was <0.05 after adjusting for multiple comparisons [55]. Genes with >1.5 fold change in expression were considered significant using parameters previously demonstrated to define significant differential expression [17].

### Quantitative Real-Time PCR (qRT-PCR)

Twelve of the 24 strains, including six from clade 8 (three with *stx2*, and three with *Stx2*, *2c*) and six from clade 2 (three with *stx2*, and three with *stx1*, *2*), were randomly selected for qRT-PCR analyses to equally represent clade and *stx* profiles of strains. MAC-T challenge experiments were repeated with these 12 strains for additional RNA extractions. cDNA synthesis, qRT-PCR reaction mix and cDNA amplification conditions were described previously [13]. Primers sequences and annealing temperatures for the 16 genes examined are in **Supplemental Table S2**; the 16S *rrsH* gene was used for within sample normalization [55]. Gene expression for each clade was calculated as an individual data point (mean ± SD of six strains), as there was no means to justify which individual strains were to be compared [58]. The relative differences in expression were presented as the ratio of clade 8 to clade 2.

### Sequence analysis

Because sequence comparisons among three O157:H7 genomes have demonstrated differences in phage gene content [5], the distribution of phage genes was determined by BLASTN [59] analysis of O157:H7 genomes from strains Sakai (clade1) [12], EDL 933 (clade 3) [60], TW14359 (clade 8) [61], and TW14588 (clade 2, GenBank Acc. No. ABKY00000000); clade membership of these strains was determined previously [5]. Phage genes that were not present in both TW14359 and TW14588 strains were omitted from the analysis.

## Supporting Information

Figure S1 Invasion of MAC-T cells by 24 O157:H7 strains. Plotted on the ordinate are invasion ratios of test strain to Sakai (y-left), as well as CFU/ml plate counts (y-right). Strains were ranked on the abscissa according to association levels for consistency with Figure 1. The symbols indicate the mean ± SD of three separate experiments. The dotted line represents the invasion level of Sakai.
(1.87 MB TIF)

Figure S2 Adherence of 24 O157:H7 strains to MAC-T cells. For each strain, adherence was calculated by subtracting invasion from association levels (CFU/ml). Plotted on the ordinate are adherence ratios of test strain to Sakai (y-left), as well as CFU/ml plate counts (y-right). Strains were ranked on the abscissa according to association levels for consistency with Figure 1. The symbols indicate the mean ± SD of three separate experiments. The dotted line represents the adherence level of Sakai.
(1.85 MB TIF)

Figure S3 Genes that were significantly differentially expressed between 4 groups (clade stx) of O157:H7 strains. Each column represents one of the 4 groups (clade stx). Genes were sorted by chromosomal positions and the heat map was generated in R (‘gplots’ package version 2.3.2). Note that dendrogram, based on column means, clustered groups according to clade. Sp - Sakai prophage, SpLE - Sakai prophage-like element, pO157 - EHEC plasmid.
(6.98 MB TIF)

Figure S4 Fluorescence micrograph of MAC-T cells co-incubated with O157:H7 strain 93-111 (clade 2) for 3 h. Filamentous actin was stained green (Alexa Fluor 488), nucleic acid was stained red (propidium iodide). Merging the green and red fluorescence demonstrated complementarity of actin pedestals and bacterial location. White scale bar represents 10 µm. Magnification 63x with 3.6x scan zoom for 93-111.
(7.53 MB TIF)

Table S1 Relative difference in expression of 604 genes between clades 8 (n = 12) and 2 (n = 12), based on Fs analysis (R/MAANOVA) of microarray data.
(0.12 MB XLS)

Table S2 Primer sequences and annealing temperatures used for qRT-PCR.
(0.05 MB DOC)
