# Supplementary figures and images for: Increased Adherence and Expression of Virulence Genes in a Lineage of Escherichia coli O157:H7 Commonly Associated with Human Infections
Source: PLoS One. 2010 Apr 21;5(4):e10167. doi: 10.1371/journal.pone.0010167 (PMC2858043; doi:10.1371/journal.pone.0010167)

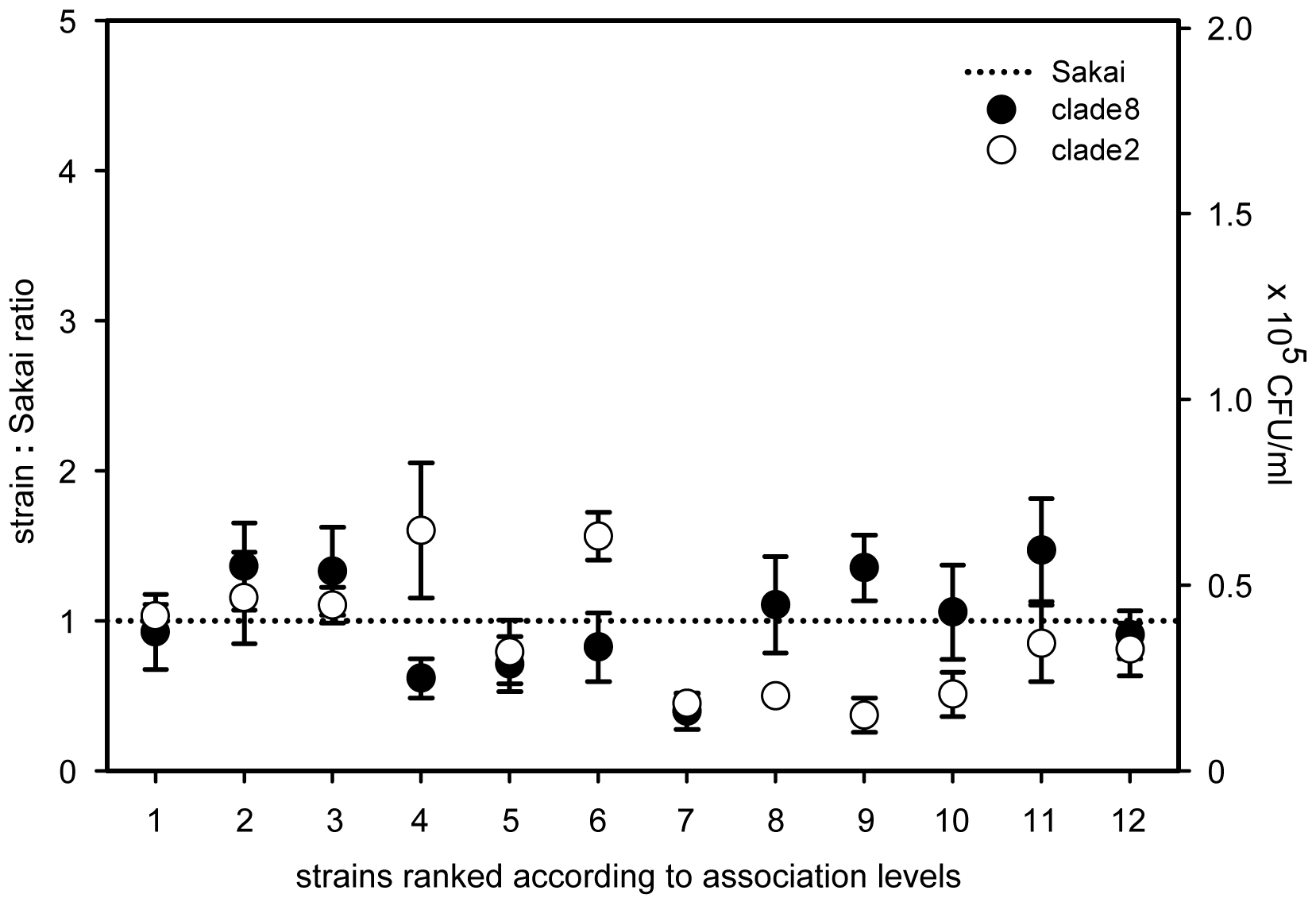

Supplement: Figure S1 — Invasion of MAC-T cells by 24 O157:H7 strains. Plotted on the ordinate are invasion ratios of test strain to Sakai (y-left), as well as CFU/ml plate counts (y-right). Strains were ranked on the abscissa according to association levels for consistency with Figure 1. The symbols indicate the mean ± SD of three separate experiments. The dotted line represents the invasion level of Sakai. (1.87 MB TIF) [file pone.0010167.s001.tif]

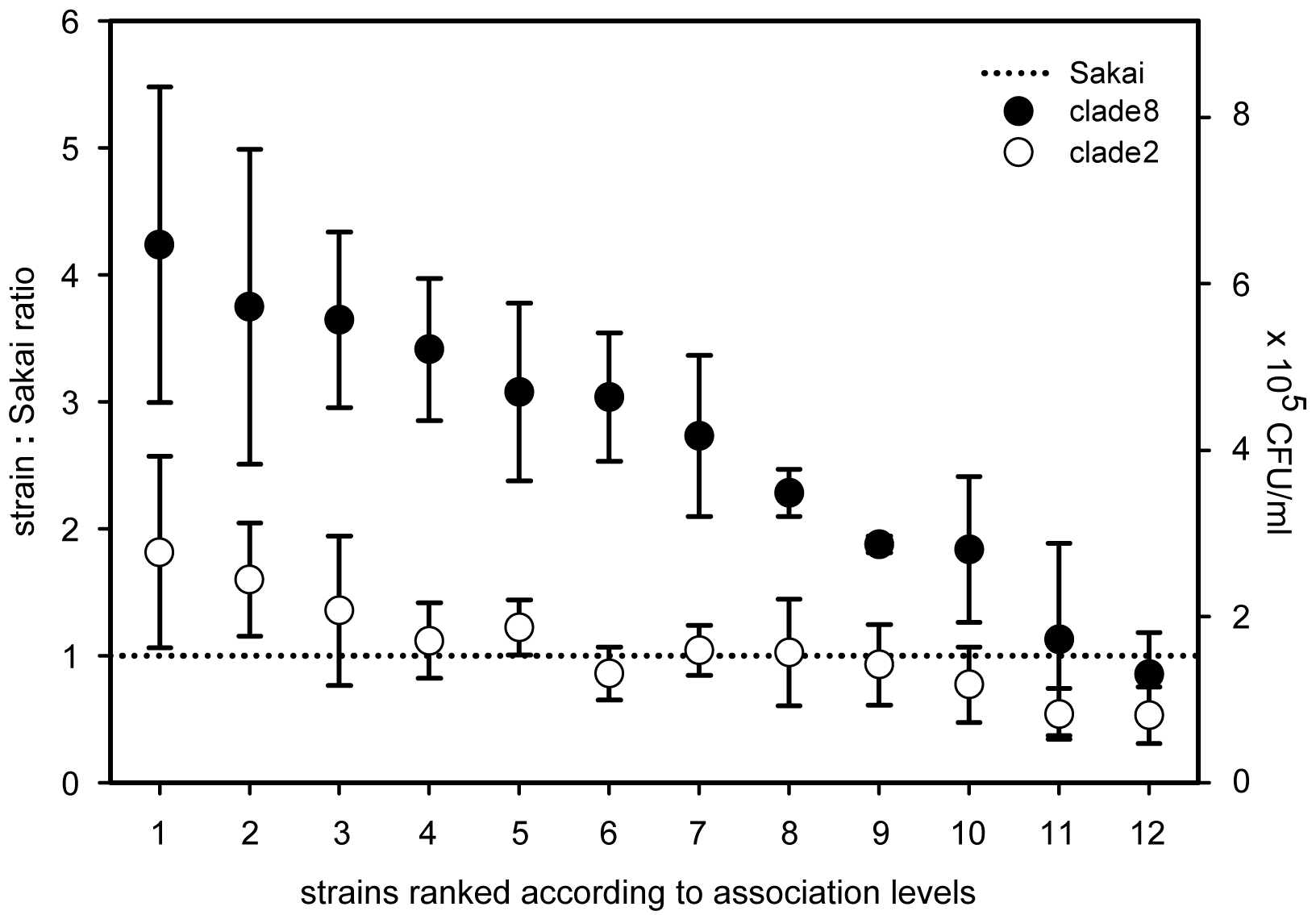

Supplement: Figure S2 — Adherence of 24 O157:H7 strains to MAC-T cells. For each strain, adherence was calculated by subtracting invasion from association levels (CFU/ml). Plotted on the ordinate are adherence ratios of test strain to Sakai (y-left), as well as CFU/ml plate counts (y-right). Strains were ranked on the abscissa according to association levels for consistency with Figure 1. The symbols indicate the mean ± SD of three separate experiments. The dotted line represents the adherence level of Sakai. (1.85 MB TIF) [file pone.0010167.s002.tif]

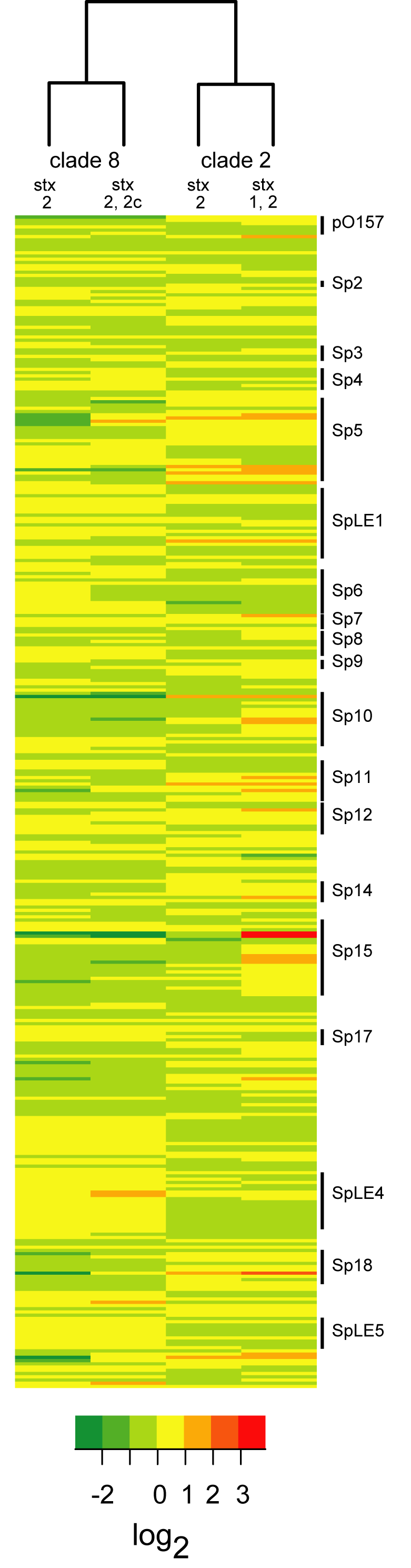

Supplement: Figure S3 — Genes that were significantly differentially expressed between 4 groups (clade stx) of O157:H7 strains. Each column represents one of the 4 groups (clade stx). Genes were sorted by chromosomal positions and the heat map was generated in R (‘gplots’ package version 2.3.2). Note that dendrogram, based on column means, clustered groups according to clade. Sp - Sakai prophage, SpLE - Sakai prophage-like element, pO157 - EHEC plasmid. (6.98 MB TIF) [file pone.0010167.s003.tif]

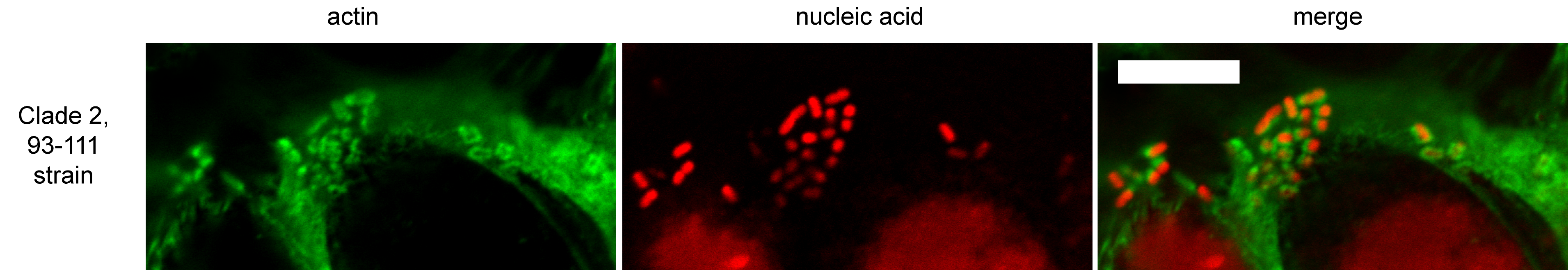

Supplement: Figure S4 — Fluorescence micrograph of MAC-T cells co-incubated with O157:H7 strain 93-111 (clade 2) for 3 h. Filamentous actin was stained green (Alexa Fluor 488), nucleic acid was stained red (propidium iodide). Merging the green and red fluorescence demonstrated complementarity of actin pedestals and bacterial location. White scale bar represents 10 µm. Magnification 63x with 3.6x scan zoom for 93-111. (7.53 MB TIF) [file pone.0010167.s004.tif]
