# Supplementary material for: Increased Adherence and Expression of Virulence Genes in a Lineage of Escherichia coli O157:H7 Commonly Associated with Human Infections
Source: PLoS One. 2010 Apr 21;5(4):e10167. doi: 10.1371/journal.pone.0010167 (PMC2858043; doi:10.1371/journal.pone.0010167)
Supplement: Table S2 — Primer sequences and annealing temperatures used for qRT-PCR. (0.05 MB DOC) [file pone.0010167.s006.doc]

**Table S2**. Primer sequences and annealing temperatures used for qRT-PCR.

| Primer |  | Sequence (5’ to 3’) | Annealing  temperature |
| --- | --- | --- | --- |
| *eae-*F | Intimin | GCCGGTAAAGCGACTGTTAG | 55ºC |
| *eae-*R |  | ATTAGGCAACTCGCCTCTGA |  |
| *tir-*F | Tir | ACTTCCAGCCTTCGTTCAGA | 57ºC |
| *tir-*R |  | TTCTGGAACGCTTCTTTCGT |  |
| *espA-*F | TTSS | gctgatgttcagagtagc | 56ºC |
| *espA-*R |  | atcaccactaagatcacg |  |
| *espB-*F | TTSS | TCAGCATTGGGGATCTTAGG | 57ºC |
| *espB-*R |  | CTGCGACATCAGCAACACTT |  |
| *grlA*-F | Regulator, TTSS | tagaaagtcctggaacaac | 56ºC |
| *grlA*-R |  | agactgtcccacaatacc |  |
| *ler*-F | Regulator, TTSS | GACTGCGAGAGCAGGAAGTT | 59ºC |
| *ler*-R |  | CAGGTCTGCCCTTCTTCATT |  |
| *escN*-F | TTSS | GATGGCATAGGCAGACCAAT | 55ºC |
| *escN*-R |  | GCCCTGACGCCAAGTATAAA |  |
| *stx2A-*F | Shiga toxin 2A | TATATCAGTGCCCGGTGTGA | 55ºC |
| *stx2A-*R |  | TGACGACTGATTTGCATTCC |  |
| *stx2B-*F | Shiga toxin 2B | GAAGATGTTTATGGCGGT | 55ºC |
| *stx2B-*R |  | CACTGTAAATGTGTCATC |  |
| *q*-F* | Q antiterminator | CTATGAGGATGTTACATGG | 56ºC |
| *q*-R |  | CAACACGTAATAATCAACC |  |

**Table S2**, continued.

| Primer |  | Sequence (5’ to 3’) | Annealing  temperature |
| --- | --- | --- | --- |
| *stx2c-*F | Shiga toxin 2c | CTGAACAGAAAGTCACAGTYTTTA | 57ºC |
| *stx2c-*R |  | GGCCACTTTTACTGTGAATGTATC |  |
| *hlyA*-F | Enterohemolysin A  pO157 | CCAGGAGAAGAAGTTAGAG | 56ºC |
| *hlyA*-R |  | CAGACCATGTATCCTTACC |  |
| *toxB*-F | ToxB  pO157 | AGAACTCCAACGCATCAGAGA | 57ºC |
| *toxB*-R |  | TGCAGGTATTCCTCCTATTGC |  |
| *tagA/stcE*-F | StcE metalloproteinase pO157 | CCAGAAGGACTTACCTATAC | 56ºC |
| *tagA/stcE*-R |  | GAAGGCTATATCCTGACC |  |
| *rpoS*-F | RpoS sigma factor 38 | TTATCGAAGAGGGCAACCTG | 55ºC |
| *rpoS*-R |  | GTTCAATCGTCTGGCGAATC |  |
| *gadX*-F | GadX acid resistance regulator | TTACAACCGAACATGCGAAC | 59ºC |
| *gadX*-R |  | CAGACTTGGACTCATCAACAGC |  |
| *rrsH*-F | 16S rRNA | CGATGCAACGCGAAGAACCT | 55ºC |
| *rrsH*-R |  | CCGGACCGCTGGCAACAAA |  |

* the forward primer for the *q* gene (ECs1203) is specific for the *q* antiterminator of the Stx2 phage
